# An Attempt to Detect siRNA-Mediated Genomic DNA Modification by Artificially Induced Mismatch siRNA in *Arabidopsis*

**DOI:** 10.1371/journal.pone.0081326

**Published:** 2013-11-21

**Authors:** Yosuke Miyagawa, Jun Ogawa, Yuji Iwata, Nozomu Koizumi, Kei-ichiro Mishiba

**Affiliations:** Graduate School of Life and Environmental Sciences, Osaka Prefecture University, Sakai, Osaka, Japan; NIGMS, NIH, United States of America

## Abstract

Although tremendous progress has been made in recent years in identifying molecular mechanisms of small interfering RNA (siRNA) functions in higher plants, the possibility of direct interaction between genomic DNA and siRNA remains an enigma. Such an interaction was proposed in the ‘RNA cache’ hypothesis, in which a mutant allele is restored based on template-directed gene conversion. To test this hypothesis, we generated transgenic *Arabidopsis thaliana* plants conditionally expressing a hairpin dsRNA construct of a mutated acetolactate synthase (*mALS*) gene coding sequence, which confers chlorsulfuron resistance, in the presence of dexamethasone (DEX). In the transgenic plants, suppression of the endogenous *ALS* mRNA expression as well as 21-nt *mALS* siRNA expression was detected after DEX treatment. After screening >100,000 progeny of the *mALS* siRNA-induced plants, no chlorsulfuron-resistant progeny were obtained. Further experiments using transgenic calli also showed that DEX-induced expression of *mALS* siRNA did not affect the number of chlorsulfuron-resistant calli. No trace of cytosine methylation of the genomic *ALS* region corresponding to the dsRNA region was observed in the DEX-treated calli. These results do not necessarily disprove the ‘RNA cache’ hypothesis, but indicate that an RNAi machinery for *ALS* mRNA suppression does not alter the *ALS* locus, either genetically or epigenetically.

## Introduction

RNA silencing is a fundamental mechanism of gene regulation in eukaryotes, which uses double-stranded RNAs or stem-loop precursor-derived 21-28 nucleotide (nt) small RNAs to guide mRNA degradation, control mRNA translation or chromatin modification [1]. Additionally, recent progress in RNA studies has unveiled uncharacterized features of non-coding RNAs. Circular RNAs were discovered recently and are thought to have a role as an effector of miRNAs [2], [3]. Certain types of RNAs may participate in DNA modifications. In the ciliate *Oxytricha*, RNA-mediated genomic rearrangement and DNA repair are observed [4]. Recent studies also suggest that small RNAs could play a role in double-stranded break (DSB) repair in yeast, plants and animals, although the detailed mechanism is not clear [5], [6], [7]. Experimental illustrations of site-specific base changes accomplished by chimeric RNA/DNA oligonucleotides in chromosomal targets [8], [9], [10] also suggest that RNA might have a function in mismatch recognition and repair.

This analogy led us to reinvestigate the previously argued ‘RNA cache’ hypothesis, which proposed a possible explanation for non-Mendelian inheritance of *hothead* (*hth*) mutants in *Arabidopsis* [11]. In the hypothesis, a wild-type *HTH* allele was obtained from the offspring of *hth* homozygotes, where a “cache” of double-stranded RNA from the *HTH* ancestors effected the reversion. This non-Mendelian inheritance phenomenon inspired several alternative explanations: gene conversion by short homologous genomic DNA sequences [12] or by supernumerary chromatin fragments propagating within meristem cells [13], mutagenesis by accumulation of mutagenic compounds in *hth* mutants [14], or production of a chimeric embryo fused with maternal cells in *hth* mutants [15]. On the other hand, subsequent examinations suggested that this non-Mendelian behavior of *hth* could be explained by their susceptibility to outcrossing [16], [17]. Although the latter explanation seems plausible, Lolle and co-authors provided additional data that *hth* mutants can spontaneously produce mosaic sectors with *HTH* alleles [18].

Apart from the argument of the RNA cache hypothesis, it would be intriguing to verify whether an RNA molecule can restore a mutated DNA sequence *in vivo*. To address this, we provided an experimental demonstration of the effect of the expression of a hypothetical RNA cache on modification of the host genome sequence. Among several types of RNA molecules, we chose double-strand RNA (dsRNA) as a template for restoring the DNA sequence, because small RNAs derived from dsRNA participate in DNA modification (e.g. DSB or RNA-dependent DNA methylation (RdDM)) in the nucleus in some cases [6], [19].

To detect sensitively a genomic DNA modification event, we chose the acetolactate synthase (*ALS*) gene [20], because a mutation in the *ALS* gene has been used for gene therapy studies [9], [10], [21], [22]. The *ALS* gene catalyzes the first step in the synthesis of branched-chain amino acids (valine, leucine, and isoleucine), and a mutation that causes an amino acid substitution at Pro-197 to Ser confers dominant resistance to the herbicide chlorsulfuron [23], [24].

In the present study, an inverted-repeat construct harboring the mutated *ALS* (*mALS*) sequence was introduced into a chemically inducible vector. Existence of chlorsulfuron-resistant transgenic *Arabidopsis* plants or calli was assessed after induction of *mALS* siRNA to determine the effect of RNA-mediated site-specific mutagenesis. We also discussed the possibility of the occurrence of RdDM simultaneously with RNAi.

## Materials and Methods

### Vector construction and transgenic plant production

The dexamethasone (DEX)-inducible RNAi binary vector, pOpOff2(hyg) was kindly provided by Dr Helliwell [25]. Genomic DNA isolated from the *Arabidopsis csr1-1* mutant [26], obtained from the Arabidopsis biological resource center (ABRC) (CS204), was used as a template to amplify the *csr1-1* locus by PCR, using primer pair, 5'-TATCCTCGTCGAAGCTCTAGAACGTCAAGGCGTAG-3' and 5'-AAGTAGCTAAAAAGAAGGCCTCCTCAATAATCCTAGGG-3'. The former primer contains a single mismatch to introduce an *Xba*I site instead of the original *Hin*dIII site; the latter primer contains two mismatches to introduce a *Stu*I site instead of the original *Hin*dIII site. The PCR product (428 bp) was cloned into the pCR8/GW/TOPO vector (Life Technologies, Carlsbad, CA) and used for the GATEWAY reaction to make pOpOff2mALSir by introducing the fragment as an inverted repeat into the pOpOff2(hyg) vector. This binary vector was transferred to *Agrobacterium tumefaciens* strain EHA101 [27] by the freeze-thaw method [28]. Stable transformation of *Arabidopsis* plants was performed using the floral dip method [29].

### Plant culture and DEX treatment

*In vitro* cultured homozygous (T3) transgenic plant seedlings at 5 days after germination were transferred to MS medium with 5 µM DEX and/or 2 mM of valine and isoleucine [in some cases, 0.1 % (w/v) casamino acids was used instead of the amino acids]. Callus was induced from young leaf tissues on 0.25% gellan gum-solidified MS medium containing 1 mg l^-1^ 2,4-D and 0.1 mg l^-1^ kinetin for 3 weeks of culture and the calli were subcultured on the same medium every 3 weeks. The same concentrations of DEX and amino acids as detailed above were used for callus treatments. Chlorsulfuron selection was performed on medium containing 100 nM chlorsulfuron.

### mRNA and siRNA expression analyses

Total RNA was extracted using the RNeasy Plant Mini kit (Qiagen, Hilden, Germany), according to the manufacturer’s protocol. Each 1 µg of RNA was reverse transcribed with random primers using a High Capacity cDNA Reverse Transcription Kit (Life Technologies), according to the manufacturer’s protocol. Real-time PCR measurements were performed using a 7300 Real-Time PCR System (Life Technologies) and SYBR Premix Ex Taq (Takara Bio, Otsu, Japan). The primers used were as follows: 5'-GGCGAGGGTGACAAAGAAAG-3' and 5'-TCTTGGTGCGGACAAATCAC-3' for *ALS* (At3g48560). Transcript abundance was normalized to the expression of *Act8* using primers as previously described [30].

Low molecular-weight RNAs were isolated from each 0.08 g FW of young leaf or callus tissues using a High Pure miRNA Isolation Kit (Roche, Basel, Switzerland). The low molecular-weight RNA samples were separated by 15% polyacrylamide gel electrophoresis (PAGE) at 180V, and the gel was transferred to a Biodyne A (PALL, Port Washington, NY) nylon membrane using a semidry blotter. After transfer, 1-ethyl-3-(3-dimethylaminopropyl) carbodiimide (EDC)-mediated cross-linking [31] was performed for 2 h at 60 °C. Hybridization and non-radioactive detection of siRNAs were performed as described previously [32]. A digoxigenin (DIG)-labeled *csr1-1* gene probe was prepared by PCR using the same primers as those used for the pOpOff2mALSir vector construction.

### Southern blotting and sequencing analysis of the *ALS* locus

For Southern blot analysis and bisulfite genomic sequencing, genomic DNAs were isolated from young leaves or callus tissues using the GenElute Plant Genomic DNA Miniprep kit (Sigma-Aldrich, St. Louis, MO), following the supplier’s instructions. *Hin*dIII-digested genomic DNAs (1 µg aliquots) were separated by electrophoresis on 0.8% (w/v) agarose gels, blotted onto nylon membranes and then fixed by UV irradiation. The blots were hybridized with a DIG-labeled *hpt* gene probe [33] and detected as previously described [34].

Sequencing of the *ALS* gene locus in the chlorsulfuron-resistant calli was performed by direct-PCR amplification from tissues. Callus tissues were wiped onto dried filter paper, scraped using the base end of a toothpick and washed into a 0.2 mL tube containing 25 µL of PCR reaction including the KOD FX Neo polymerase (TOYOBO, Osaka, Japan), following the supplier’s instructions. Primers used for the *ALS* locus were as follows: 5'-CCAAACCCGAAACATTCATC-3' and 5'-GAATCGCAAGCTGTTGTTGA-3', where both of the primers are located outside the *ALS* inverted-repeat region of the pOpOff2mALSir vector. The PCR reaction was performed at 94 °C for 3 min; followed by 40 cycles of 98 °C for 11 s, 60 °C for 30 s and 68 °C for 1 min. PCR products were purified with ExoSAP-IT (GE Healthcare, Little Chalfont, UK) and sequenced with the same primers used for PCR.

### Bisulfite genomic sequencing

Bisulfite genomic sequencing [35] was performed using an EpiTect Bisulfite Kit (Qiagen), as previously described [36]. Primers used for the amplification of the *ALS* gene coding region (306 bp) from sodium bisulfite-treated DNA templates were 5'-TTAGYGGATTAGYYGATGYGTTGTTAGATAGTG-3' and 5'-ACATATAACCARRTAATCTCATARCCTRTTCCC-3'. PCR products were cloned into vector pSTBlue-1 (Novagen, Madison, WI) and 16 clones from each sample were independently sequenced. The sequence data were applied to CyMATE [37] (http://www.gmi.oeaw.ac.at/research-groups/cymate) to identify methylated cytosine sequences.

## Results

### Production of transgenic *Arabidopsis* plants conditionally expressing *mALS* dsRNA

**Figure 1:**
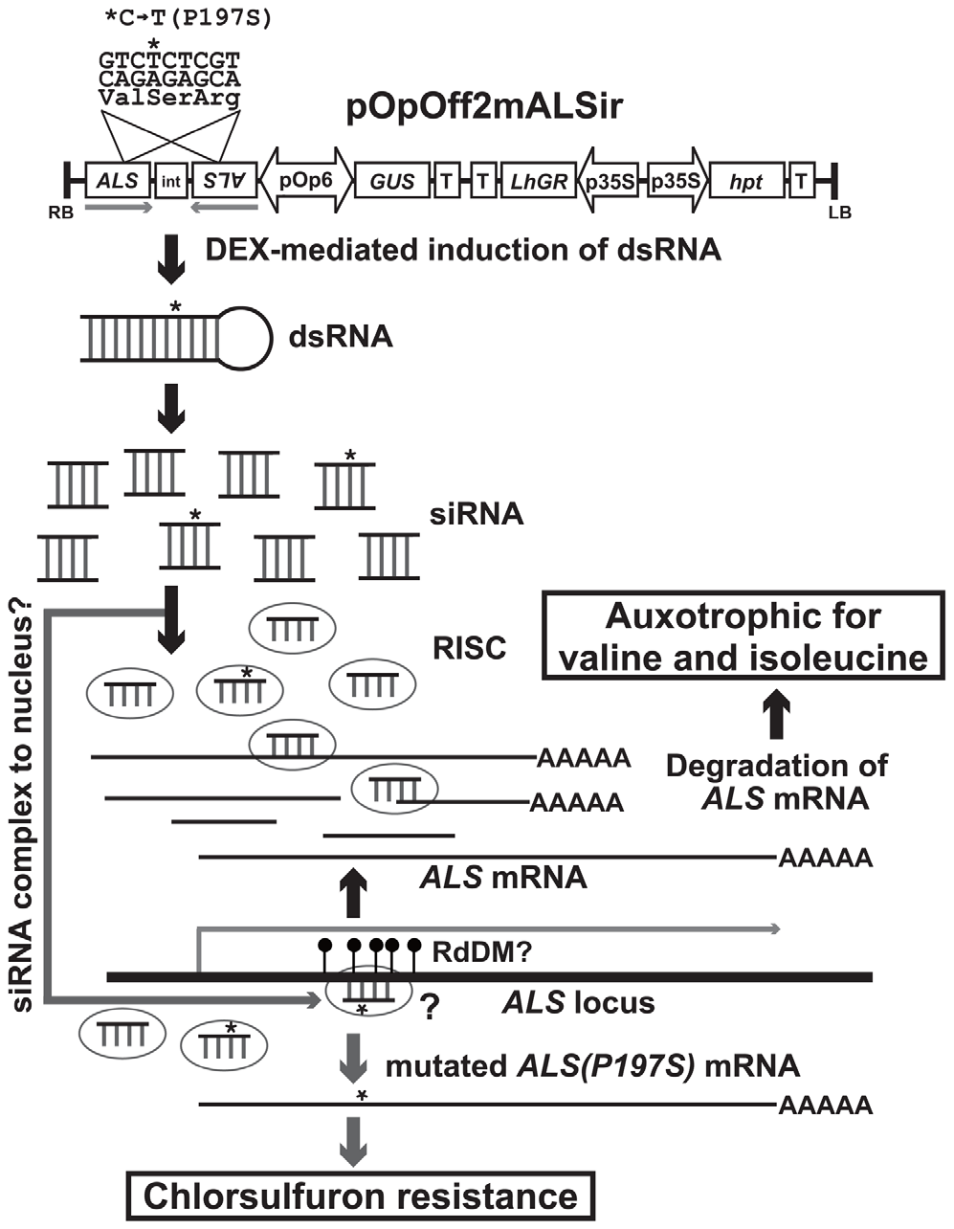
Schematic diagram of mutated acetolactate synthase (*mALS*) dsRNA induction vector and possible consequence of endogenous *ALS* mRNA degradation and hypothetical modification of the genomic *ALS* sequence. The *mALS* dsRNA is transcribed from the pOpOff2mALSir binary vector (upper) after dexamethasone (DEX) treatment, which activates LhGR transcription factor targeting the pOp6 bidirectional promoter. The *mALS* dsRNA is processed into siRNAs, which in turn compose an RNA-induced silencing complex (RISC) and the endogenous *ALS* mRNA is expected to be degraded by the RNAi machinery. At the same time, accumulation of *mALS* siRNA may be guided to the complementary genomic *ALS* locus (lower), resulting in *ALS* mutation, which would confer chlorsulfuron resistance. The black circles on the *ALS* locus represent hypothetical DNA methylation caused by RNA-dependent DNA methylation (RdDM).

To construct a hypothetical RNA substrate for DNA modification in *Arabidopsis* tissues, an inducible RNAi binary vector, pOpOff2(Hyg) [25], was used. This vector expresses a CaMV 35S promoter-driven synthetic transcription factor from the *LhGR* gene, which activates the pOp6 promoter within the vector by association with a synthetic glucocorticoid, DEX. The bidirectional pOp6 promoter drives both the beta-glucuronidase (GUS) gene and an inverted-repeat partial (428-bp) *mALS* cDNA sequence with an intron (Figure 1). The *mALS* sequence, derived from a chlorsulfuron-resistant *csr1-1* mutant [24], contains a point mutation (C589T) leading to the amino acid substitution P197S. After DEX treatment, this inverted-repeat *mALS* transcript is expected to produce dsRNA after removal of the intron, followed by siRNA processing by Dicer-like (DCL) enzymes and incorporated in a RISC (Figure 1). In this study, we examined whether the artificially expressed *mALS* small RNA could alter the genomic sequence of the corresponding *ALS* locus.

**Figure 2:**
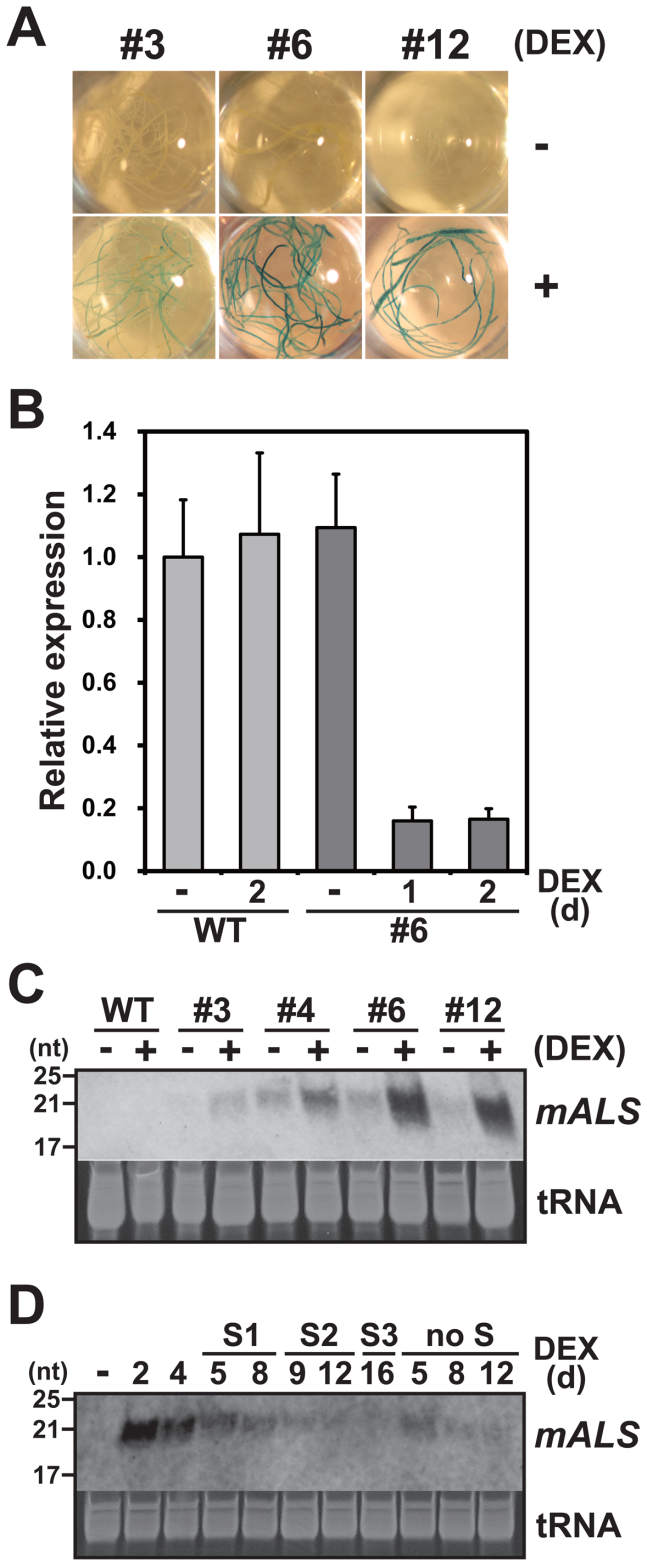
Phenotypes of transgenic *Arabidopsis* plants expressing *mALS* siRNA after DEX treatment. (A) Beta-glucuronidase (GUS) expression in root tissues of the transgenic plant lines #3, #6 and #12 after treatment with (+) or without (-) DEX. (B) Relative mRNA expression of the *ALS* gene in the wild-type (WT) and transgenic (#6) plants with or without (-) DEX treatment. *ALS* mRNA expression was normalized to the expression level of *Act8*. Experiments were replicated three times. (C) Expression of siRNA derived from *mALS* dsRNA in the transgenic plants (lines #3, #4, #6 and #12) after DEX treatment. DEX was applied for 2 days. Ethidium bromide staining of tRNA is shown as a loading control. (D) Effect on *mALS* siRNA expression of subculturing onto medium containing DEX in the #6 transgenic plants. Plants cultured after one (S1) to three (S3) rounds of subculturing or without subculturing (no S) for different periods (indicated as days after treatment) were analyzed.

A number of transgenic *Arabidopsis* plants harboring the inverted-repeat *mALS* construct (*mALS*ir) described above were obtained after *Agrobacterium*-mediated transformation, and single copy transgenic lines were selected using Southern blotting and segregation analyses. As a consequence, four independent transgenic lines (#3, #4, #6 and #12) were selected and used for further experiments. All four lines showed GUS expression in their root tissues, with three lines #3, #6 and #12 showing strong expression (Figure 2A) and line #4 showing weaker expression (data not shown), only when cultured on DEX-containing medium, suggesting DEX-dependent induction of the pOp6 promoter. We then examined knockdown of the endogenous *ALS* gene after DEX treatment in the transgenic plants. Real-time PCR indicated a significant reduction in *ALS* mRNA expression 1 day after DEX treatment in line #6 plant tissues; no effect of DEX treatment on *ALS* expression was observed in non-transgenic plants (Figure 2B). Consistently, DEX treatment, which barely affected the growth of the non-transgenic plants, caused the transgenic plants to wither (Figure S1). This growth inhibition was mitigated by the addition of valine and isoleucine, indicating downregulation of ALS function in the DEX-treated transgenic plants (Figure S1). Acute induction of *mALS* siRNA was also demonstrated after DEX treatment in lines #6 and #12, while slight induction was observed in lines #3 and #4 (Figure 2C). The expression of *mALS* siRNA started to decline 4 days after DEX treatment, with only weak expression being detected at 8 days or later (Figure 2D). The attenuation of the *mALS* expression was not fully recovered by subculturing on fresh DEX-containing medium (Figure 2D).

### Chlorsulfuron selection of the progeny of the DEX-treated transgenic plants

*mALS* siRNA induction of the transgenic plant lines #3, #4, #6 and #12 was performed by culturing the seedlings on medium containing amino acids and DEX. After 1 month of culture, the transgenic plants were acclimatized in pots with soil and self-fertilized. From the transgenic plants treated with or without DEX, approximately 108,000 seeds were obtained, which were sown on chlorsulfuron-containing medium to select chlorsulfuron-resistant plants. Consequently, no chlorsulfuron-resistant seedlings were obtained from the progeny of DEX-treated plants or untreated plants (Table 1).

**Table 1:** Selection of chlorsulfuron-resistant selfed progeny of the transgenic plants treated with or without DEX.

Line	DEX	Treated seeds	CS^S^	CS^R^
#3	-	13,000	13,000	0
	+	25,000	25,000	0
#4	-	22,000	22,000	0
	+	38,000	38,000	0
#6	-	5,000	5,000	0
	+	23,000	23,000	0
#12	-	5,000	5,000	0
	+	22,000	22,000	0
Total	-	45,000	45,000	0
	+	108,000	108,000	0

Seeds were sown on MS medium containing 100 nM chlorsulfuron. CS^S^, number of chlorsulfuron-sensitive seedlings. CS^R^, number of chlorsulfuron-resistant seedlings.

### Chlorsulfuron selection of the DEX-treated transgenic callus

While the above-mentioned results indicated that expression of the *mALS* siRNA in *Arabidopsis* tissues does not affect the genomic DNA sequence of the *ALS* locus, there still remained the possibility that the DEX-treated transgenic plants failed to produce the *mALS* siRNA within germ-line cells. In addition, duration of the *mALS* siRNA expression seemed to be limited in the DEX-treated transgenic plant tissues (see Figure 2D), and therefore siRNA accumulation might be insufficient to modify the *ALS* genomic sequence. To address these issues, we used transgenic calli as the materials of *mALS* siRNA expression and subsequent chlorsulfuron selection.

**Figure 3:**
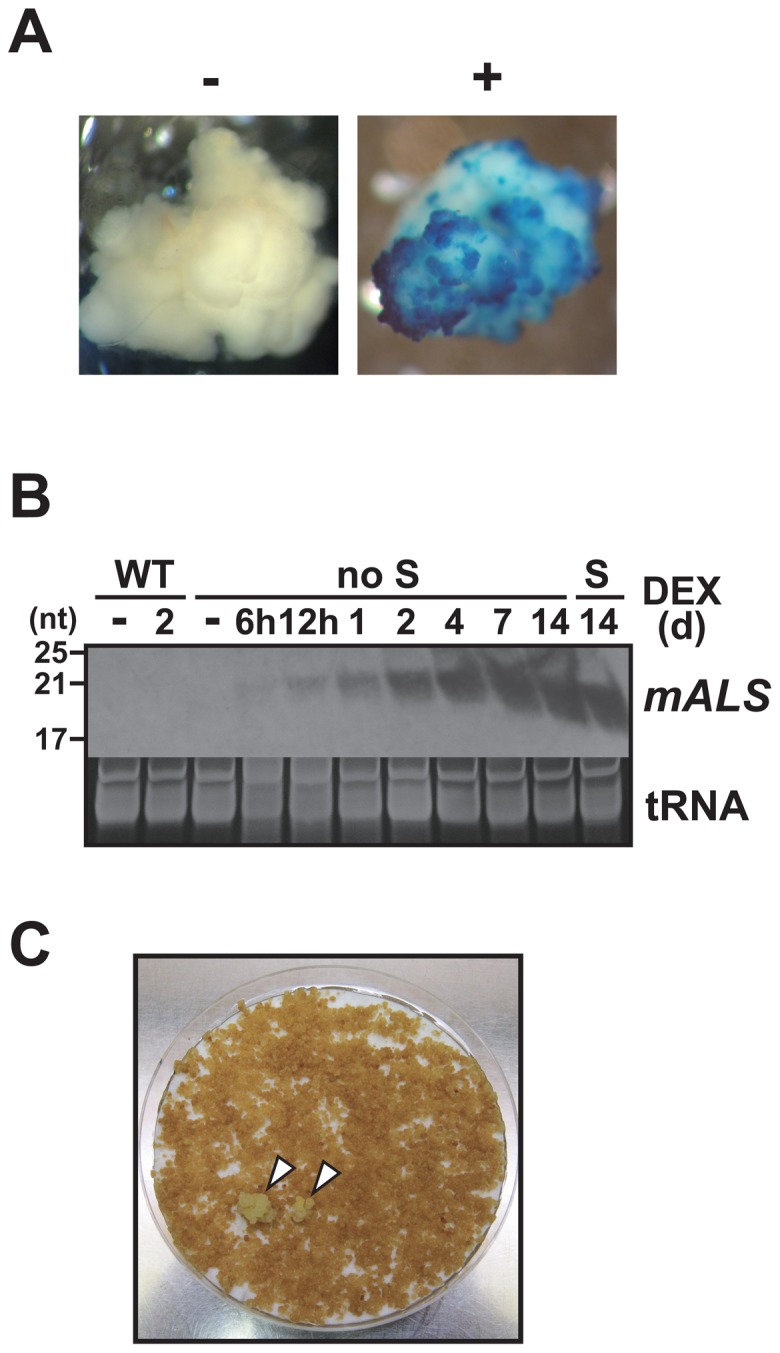
*mALS* siRNA induction and chlorsulfuron selection of the transgenic calli. (A) GUS expression of the transgenic callus line #3 after treating with (+) or without (-) DEX. (B) Stability of *mALS* siRNA expression in the transgenic callus #3 or wild-type (WT) callus on the DEX-containing medium. Calli (#3) cultured for different periods on DEX-containing medium with (S) or without (no S) subculturing after 7 days of culture were analyzed. (C) Selection of chlorsulfuron-resistant calli after 3 months of culture with medium containing 100 nM chlorsulfuron.

Calli were derived from shoot tissues of DEX-untreated transgenic lines #3, #6 and #12, as well as those of a wild-type (WT) plant as a control. GUS expression of the transgenic calli was observed after DEX treatment, whereas untreated transgenic calli and WT callus did not exhibit GUS expression (Figure 3A). The transgenic calli, but not WT callus, also expressed the *mALS* siRNA after DEX treatment. For example, in line #3 calli, *mALS* siRNA expression was detected 6 h after DEX treatment, increased over the following several days, and was sustained for at least 14 d after DEX treatment (Figure 3B), unlike in the transgenic seedlings (Figure 2D). The calli from the other lines (#6 and #12) also showed strong *mALS* siRNA expression (data not shown).

Large amounts of cells (each > 20 gFW callus) of transgenic (#3, #6, and #12) and WT calli were treated with or without DEX by culturing onto plates for 7 d, followed by transfer to medium containing chlorsulfuron. While most of the calli ceased to proliferate on the chlorsulfuron medium after 3 weeks of culture (Figure S2A), a number of chlorsulfuron-resistant colonies were observed, regardless of the DEX treatment (Figure 3C, Table 2). There was no significant difference in the number of chlorsulfuron-resistant colonies per gram FW callus between DEX-treated and untreated samples. In addition, chlorsulfuron-resistant colonies were also observed for WT calli with the same frequencies as those for the transgenic callus lines (Table 2), suggesting that there was no significant effect of the transgene sequence on chlorsulfuron-resistant colony generation.

**Table 2:** Effect of DEX treatment on the number of chlorsulfuron-resistant colonies in the transgenic calli.

Line	DEX	Treated cells (gFW)	CS^R^	Ratio (CS^R^/g)
#3	-	29.0	26	0.90
	+	5.0	2	0.40
#6	-	17.0	9	0.53
	+	7.5	4	0.53
#12	-	21.0	12	0.57
	+	8.5	6	0.70
WT	-	62.0	47	0.76

CS^R^, number of chlorsulfuron-resistant colonies.

To verify whether the generation of the chlorsulfuron-resistant colonies was caused by point mutation of the *ALS* locus, the genomic sequences of the *ALS* locus of the chlorsulfuron-resistant colonies were analyzed. Consequently, no sequence alteration in the targeted *ALS* sequence was found in any the 38 analyzed colonies derived from DEX-treated or untreated #3, #6 and #12 transgenic and WT calli, except that one WT callus showed a presumably mutated signal at the 589^th^ cytosine (Figure S2B). This result suggested that a natural mutation of the target sequence (C589T) occurs rarely in these culture conditions, despite the fact that a number of chlorsulfuron-resistant colonies were generated.

### No DNA methylation of the *ALS* coding region was found in the *mALS* siRNA expressed calli

Although there was no evidence for genomic DNA modification by the *mALS* siRNA expression, it was unclear whether a part of the siRNA complex was involved in RdDM. To investigate the effect of *mALS* siRNA expression on *de novo* DNA methylation, we performed bisulfite genomic sequencing on the endogenous *ALS* locus. Genomic DNAs isolated from the transgenic (lines #3, #6, and #12) and WT calli treated with or without DEX for 14 days were subjected to sodium bisulfite conversion followed by PCR amplification of the 306-bp of *ALS* coding region, which contains the *mALS*ir transgene region (203 bp) (Figure S3, red squares). The reverse primer of the bisulfite PCR amplification is outside of the *mALS*ir region; therefore, only the endogenous *ALS* sequence could be amplified. The analyzed region contains nine CG cytosines, seven CHG cytosines, and 30 CHH cytosine sequence contexts. Among the cytosines, eight, three, and 21 cytosines of CG, CHG and CHH sites, respectively, overlap the *mALS*ir region. Figure S3 shows the representative statuses of methylated cytosines in CG, CHG, and CHH sites of the *ALS* sequence, where columns display methylation statuses from different cells. In the WT callus, no methylation was detected in the analyzed *ALS* region, irrespective of the DEX treatment, indicating that the region does not undergo cytosine methylation in nature and that DEX treatment itself does not affect *de novo* methylation (Figure S3A). Neither untreated nor DEX-treated calli showed any cytosine methylation in any transgenic lines (Figure S3B-D), indicating that the expression of *mALS* siRNA does not affect *de novo* methylation of the corresponding *ALS* sequence.

## Discussion

There has been no experimental evidence of RNA molecules that are responsible for the reversion of the *hth* gene so far, which complicates the argument around the ‘RNA cache’ hypothesis. One possible solution is to use an experimentally introduced hypothetical ‘RNA cache’ substance. From this viewpoint, we constructed transgenic *Arabidopsis* that could induce detectable amounts of an RNA substance for possible gene conversion. We chose the *ALS* gene as a target for gene conversion because a single nucleotide substitution of *ALS* can confer dominant resistance to chlorsulfuron, providing high screening efficiency [9], [38]. Reduction of the endogenous *ALS* mRNA (Figure 2B) by RNAi machinery resulted in auxotrophy for amino acids in the transgenic plants (Figure S1), confirming that the siRNA induction system was functional. Although this inducible RNAi system sufficed for functional disturbance of ALS, expression of *mALS* siRNA decreased over time and the effect of DEX treatment was not renewed by subculturing on new medium (Figure 2D). A previous study using the same vector showed that siRNA expression level varied among transgenic lines [25]; therefore, our observation may be transgenic line-dependent, such as a position effect.

We screened progeny of the *mALS* siRNA-induced and uninduced plants on chlorsulfuron-containing medium to investigate the effect of *mALS* expression on the generation of chlorsulfuron-resistant (i.e. mutation of *ALS* at C589T) mutants. Despite screening over 100,000 seedlings, no chlorsulfuron-resistant plants were identified, irrespective of *mALS* expression (Table 1), suggesting that the ectopic expression of *mALS* siRNA does not cause *ALS* mutation *in planta*.

Induction of *mALS* siRNA expression in transgenic calli was also performed to overcome the limitation of the experiment using transgenic plants, which might fail to produce *mALS* siRNA in germ-line cells. As shown in Figure 3, abundant *mALS* siRNA expression was observed in the transgenic calli for an extended period (at least 14 days), during which time visible growth of the calli could be seen. This indicates that the callus tissues express *mALS* siRNA through cell proliferation and a cell undergoing *ALS* (C589T) mutation would be readily obtained as a chlorsulfuron-resistant colony. Considering these observations, the *mALS* siRNA expression followed by chlorsulfuron selection in the transgenic calli probably has the potential to detect a mutation in the *ALS* gene, even if it occurred rarely. Accordingly, we obtained a number of chlorsulfuron-resistant colonies, not only from DEX-treated transgenic calli, but also from WT callus, with the rates ranging from 0.40 to 0.90 resistant colonies per gram FW cells. Transgene integration and DEX treatment did not significantly affect the rate of occurrence of the resistant colonies. In addition, most of the resistant calli did not carry the point mutation of the target sequence (C589T), which might be due to non-target-site resistance [39]. Therefore, we concluded that the *mALS* siRNA expression does not represent a substance capable of *ALS* mutation under the conditions used in the present study.

To argue a point of accessibility of the siRNA complex to the corresponding genomic sequence, the present result suggests an important implication. That is, expression of the *mALS* siRNA did not affect the cytosine methylation status of the corresponding *ALS* locus. Although several experiments showed that RNAi may occur together with RdDM [40], [41], [42], the combined regulation of the RNAi and RdDM machineries are not fully understood. In the present study, 21-nt, but not 24-nt, siRNAs were found after induction of *mALS* inverted-repeat transcript, suggesting that the precursor *mALS* dsRNA might be infrequently processed by DCL3 but predominantly processed by DCL4 or DCL2 [43]. Recent studies suggest that ARGONAUTE4 (AGO4) and 24-nt siRNA complex guides RNA polymerase V (Pol V) to target loci through base pairing of the associated siRNAs [44]. Although it is still unclear whether AGO4-incorporated siRNAs associate with nascent transcripts of Pol V or genomic DNA [45], so far as is known, this type of siRNA is the most plausible RNA substance for genomic DNA modification. Under the present experimental conditions, therefore, we speculated that the *mALS* siRNA did not locate to the nucleus, probably because of the lack of DCL3-mediated processing.

How could *mALS* dsRNA be processed by DCL3 and incorporated into AGO4? Although little is known about how different DCL proteins share a dsRNA in plant tissues, DCL2, DCL3 and DCL4 are functionally redundant [46]: the sizes of siRNAs derived from a transgene were altered in different *dcl* mutants [47], [48]. Therefore, it may be possible to bring the *mALS* dsRNA to the RdDM machinery in a transgenic plant with a *dcl2/4* mutant background.

In summary, the effect of the ectopic expression of the artificial mismatch siRNA on genomic DNA modification was investigated using efficient selection of the dominant *ALS* mutation conferring herbicide resistance. Despite several attempts at *mALS* siRNA expression and subsequent selection of chlorsulfuron-resistant plants or calli, no evidence of the *ALS* modification by *mALS* siRNA expression was found. These results, *per se*, do not prove the non-existence of the ‘RNA cache’, but indicate that siRNA machinery targeting an endogenous mRNA do not genetically or epigenetically contribute to corresponding genomic DNA modification simultaneously, even in cells expressing significant amounts of the siRNA.

## Supporting Information

Figure S1 **Silencing of** ***ALS*** **by DEX treatment in the transgenic plants #6.** Wild-type (WT) or transgenic plants were germinated on medium containing DEX with (+) or without (-) 2 mM valine and isoleucine (AA), and photographed after 1 (1w) to 3 (3w) weeks.
(PDF)

Figure S2 **Chlorsulfuron selection culture and the** ***ALS*** **sequence of the chlorsulfuron-resistant callus.** (A) Wild-type callus cultured on medium with (+) or without (-) 100 nM chlorsulfuron (CS) for 1 to 3 weeks. (B) Minor base substitution profile (arrowhead) at the 589^th^ cytosine to thymine of the *ALS* gene genomic sequence derived from chlorsulfuron-resistant wild-type callus.
(PDF)

Figure S3 **Effect of** ***mALS*** **siRNA expression on** ***de novo*** **methylation of the genomic** ***ALS*** **locus in wild-type and transgenic calli.** Methylation statuses of the wild-type (WT; A) and the transgenic callus lines #3 (B), #6 (C), and #12 (D) treated with (+) or without (-) DEX for 14 d were analyzed by bisulfite genomic sequencing. Methylated sites are filled symbols for CG sites (red circles), CHG sites (blue squares), and CHH sites (green triangles). The first column (indicated as ALS) is the reference *ALS* sequence and the subsequent columns are the methylation profiles derived from different cells. Red square boxes indicate regions corresponding to the *mALS* dsRNA sequence.
(PDF)
