# Supplementary material for: An Attempt to Detect siRNA-Mediated Genomic DNA Modification by Artificially Induced Mismatch siRNA in Arabidopsis
Source: PLoS One. 2013 Nov 21;8(11):e81326. doi: 10.1371/journal.pone.0081326 (PMC3837478; doi:10.1371/journal.pone.0081326)
Supplement: Figure S1 — Silencing of ALS by DEX treatment in the transgenic plants #6. Wild-type (WT) or transgenic plants were germinated on medium containing DEX with (+) or without (-) 2 mM valine and isoleucine (AA), and photographed after 1 (1w) to 3 (3w) weeks. (PDF) [file pone.0081326.s001.pdf]

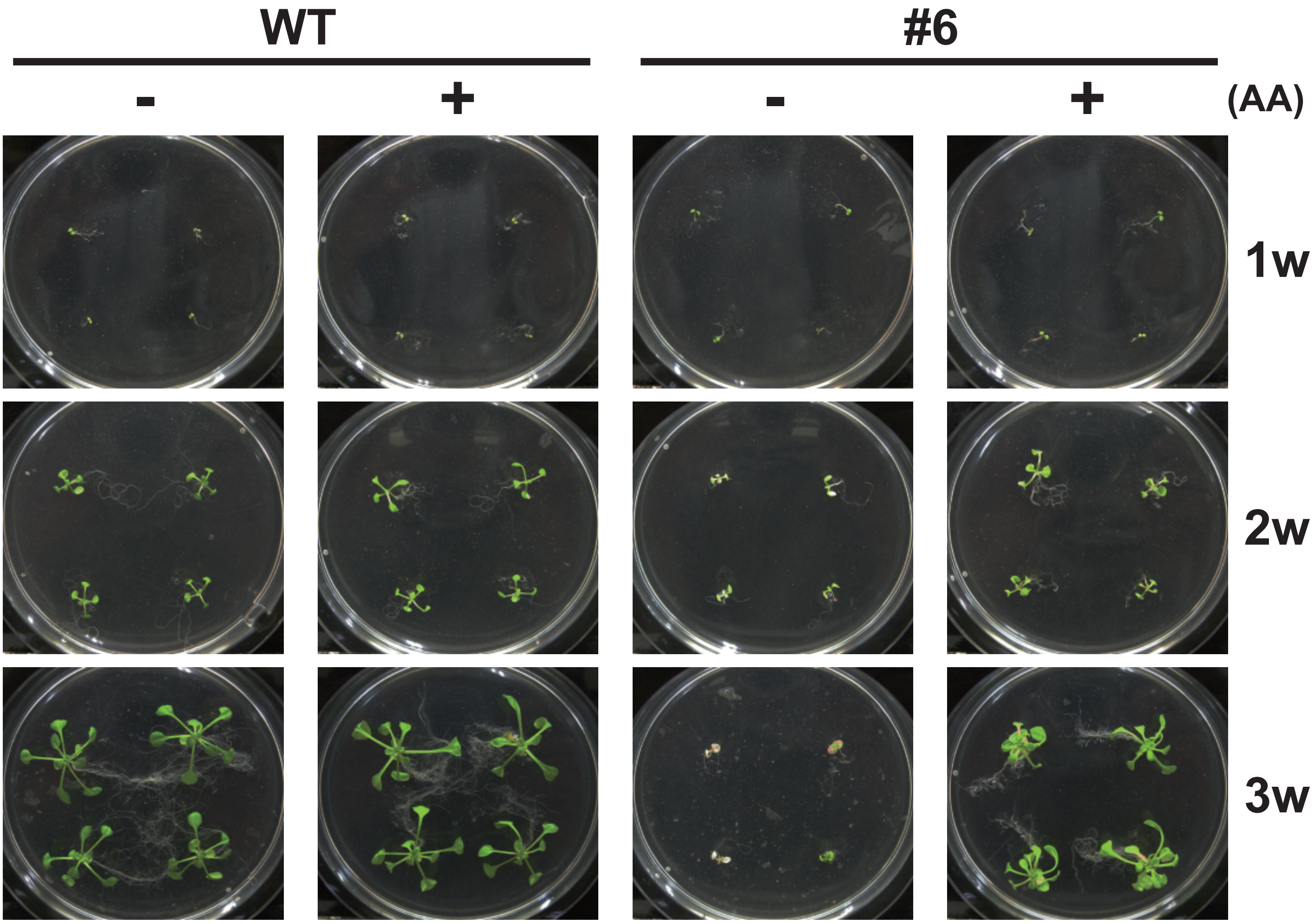

Figure S1. Silencing of *ALS* by DEX treatment in the transgenic plants #6. Wild-type (WT) or transgenic plants were germinated on medium containing DEX with (+) or without (-) 2 mM valine and isoleucine (AA), and photographed after 1 (1w) to 3 (3w) weeks.
