# Supplementary material for: An Attempt to Detect siRNA-Mediated Genomic DNA Modification by Artificially Induced Mismatch siRNA in Arabidopsis
Source: PLoS One. 2013 Nov 21;8(11):e81326. doi: 10.1371/journal.pone.0081326 (PMC3837478; doi:10.1371/journal.pone.0081326)
Supplement: Figure S2 — Chlorsulfuron selection culture and the ALS sequence of the chlorsulfuron-resistant callus. (A) Wild-type callus cultured on medium with (+) or without (-) 100 nM chlorsulfuron (CS) for 1 to 3 weeks. (B) Minor base substitution profile (arrowhead) at the 589th cytosine to thymine of the ALS gene genomic sequence derived from chlorsulfuron-resistant wild-type callus. (PDF) [file pone.0081326.s002.pdf]

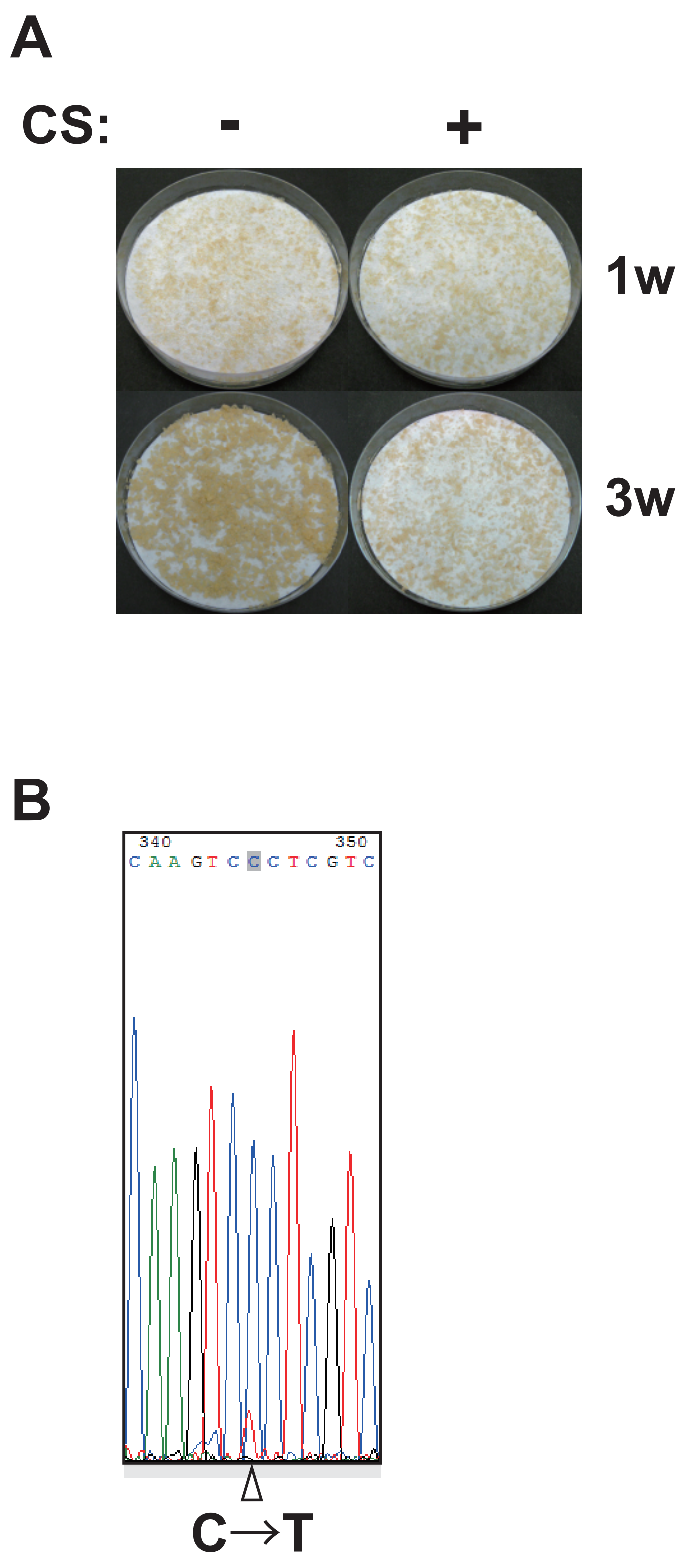

Figure S2. Chlorsulfuron selection culture and the *ALS* sequence of the chlorsulfuron-resistant callus. (A) Wild-type callus cultured on medium with (+) or without (-) 100 nM chlorsulfuron (CS) for 1 to 3 weeks. (B) Minor base substitution profile (arrowhead) at the 589th cytosine to thymine of the *ALS* gene genomic sequence derived from chlorsulfuron-resistant wild-type callus.
