# Supplementary material for: An Attempt to Detect siRNA-Mediated Genomic DNA Modification by Artificially Induced Mismatch siRNA in Arabidopsis
Source: PLoS One. 2013 Nov 21;8(11):e81326. doi: 10.1371/journal.pone.0081326 (PMC3837478; doi:10.1371/journal.pone.0081326)
Supplement: Figure S3 — Effect of mALS siRNA expression on de novo methylation of the genomic ALS locus in wild-type and transgenic calli. Methylation statuses of the wild-type (WT; A) and the transgenic callus lines #3 (B), #6 (C), and #12 (D) treated with (+) or without (-) DEX for 14 d were analyzed by bisulfite genomic sequencing. Methylated sites are filled symbols for CG sites (red circles), CHG sites (blue squares), and CHH sites (green triangles). The first column (indicated as ALS) is the reference ALS sequence and the subsequent columns are the methylation profiles derived from different cells. Red square boxes indicate regions corresponding to the mALS dsRNA sequence. (PDF) [file pone.0081326.s003.pdf]

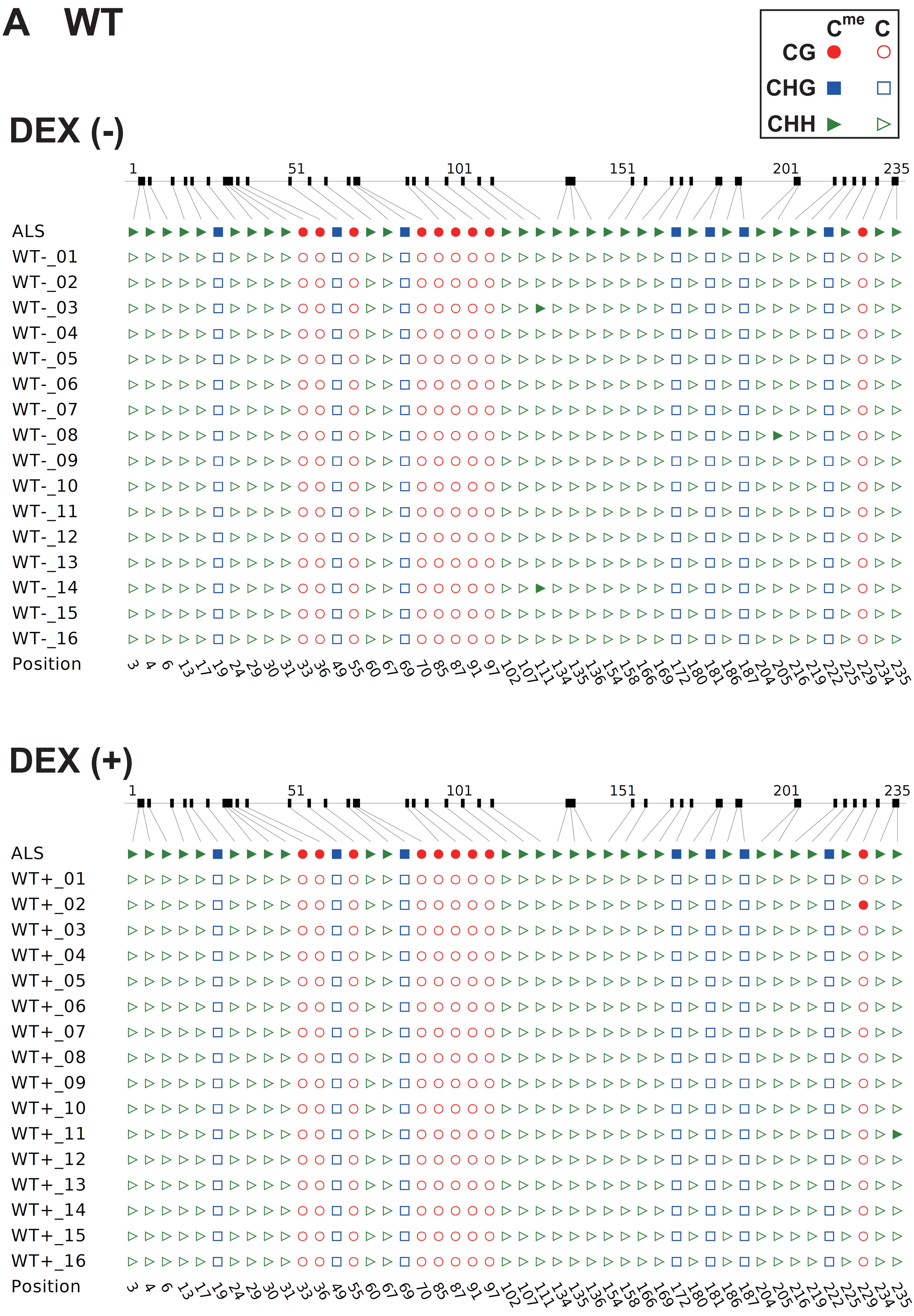
A WT
Cme C
CG
CHG
CHH
DEX (-)
1 51 101 151 201 235
ALS
WT-_01
WT-_02
WT-_03
WT-_04
WT-_05
WT-_06
WT-_07
WT-_08
WT-_09
WT-_10
WT-_11
WT-_12
WT-_13
WT-_14
WT-_15
WT-_16
Position
3 4 6 13 17 19 24 29 30 31 33 36 49 55 60 67 69 70 85 87 91 97 102 107 111 134 135 136 154 158 166 169 172 180 181 186 187 204 205 216 219 222 225 229 234 235
DEX (+)
1 51 101 151 201 235
ALS
WT+_01
WT+_02
WT+_03
WT+_04
WT+_05
WT+_06
WT+_07
WT+_08
WT+_09
WT+_10
WT+_11
WT+_12
WT+_13
WT+_14
WT+_15
WT+_16
Position
3 4 6 13 17 19 24 29 30 31 33 36 49 55 60 67 69 70 85 87 91 97 102 107 111 134 135 136 154 158 166 169 172 180 181 186 187 204 205 216 219 222 225 229 234 235

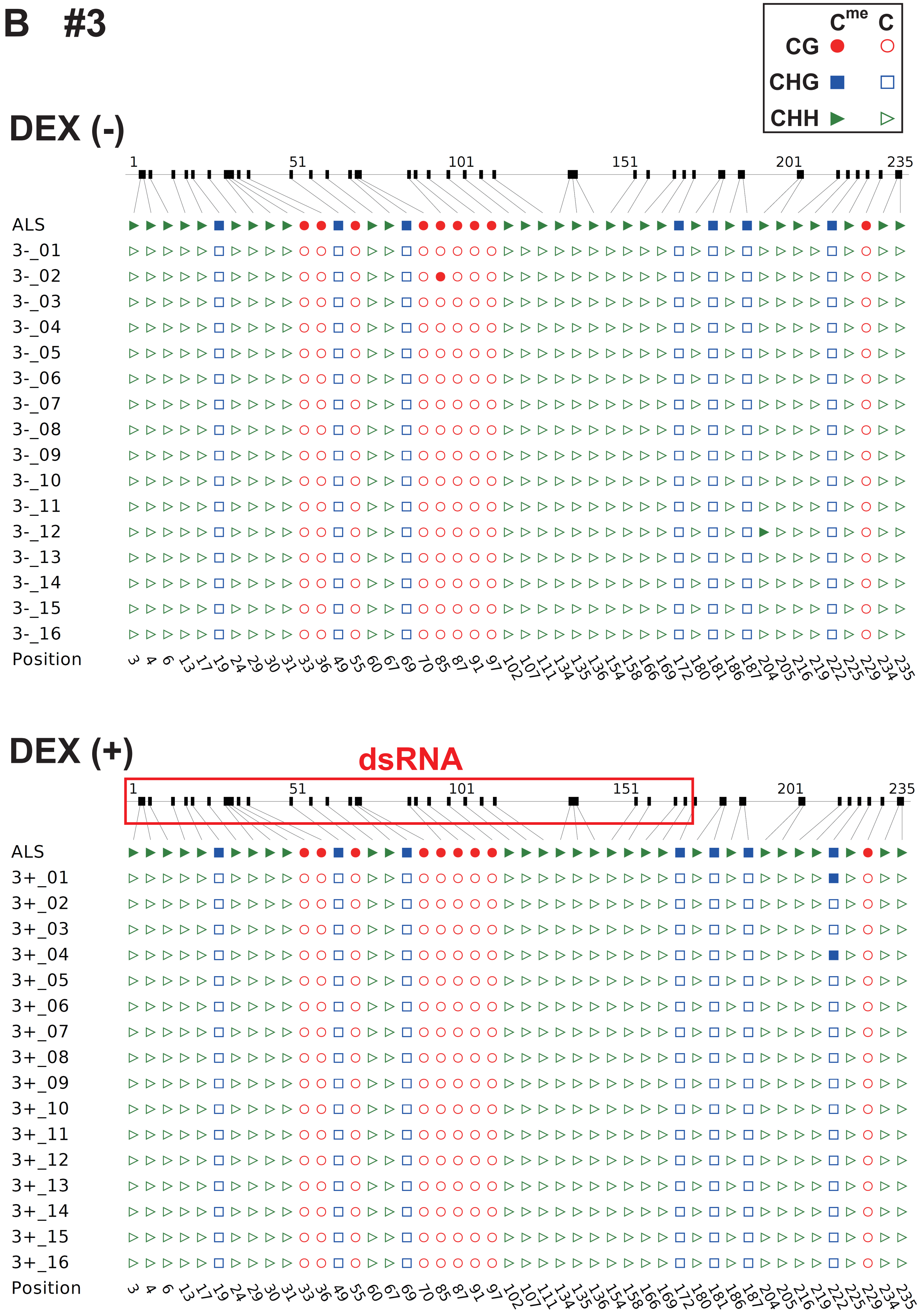
B #3
Cme C
CG
CHG
CHH
DEX (-)
1 51 101 151 201 235
ALS
3-_01
3-_02
3-_03
3-_04
3-_05
3-_06
3-_07
3-_08
3-_09
3-_10
3-_11
3-_12
3-_13
3-_14
3-_15
3-_16
Position
3 4 6 13 17 19 24 29 30 31 33 36 49 55 60 67 69 70 85 87 91 97 102 107 111 134 135 136 154 158 166 169 172 180 181 186 187 204 205 216 219 222 225 229 234 235
DEX (+)
dsRNA
1 51 101 151 201 235
ALS
3+_01
3+_02
3+_03
3+_04
3+_05
3+_06
3+_07
3+_08
3+_09
3+_10
3+_11
3+_12
3+_13
3+_14
3+_15
3+_16
Position
3 4 6 13 17 19 24 29 30 31 33 36 49 55 60 67 69 70 85 87 91 97 102 107 111 134 135 136 154 158 166 169 172 180 181 186 187 204 205 216 219 222 225 229 234 235

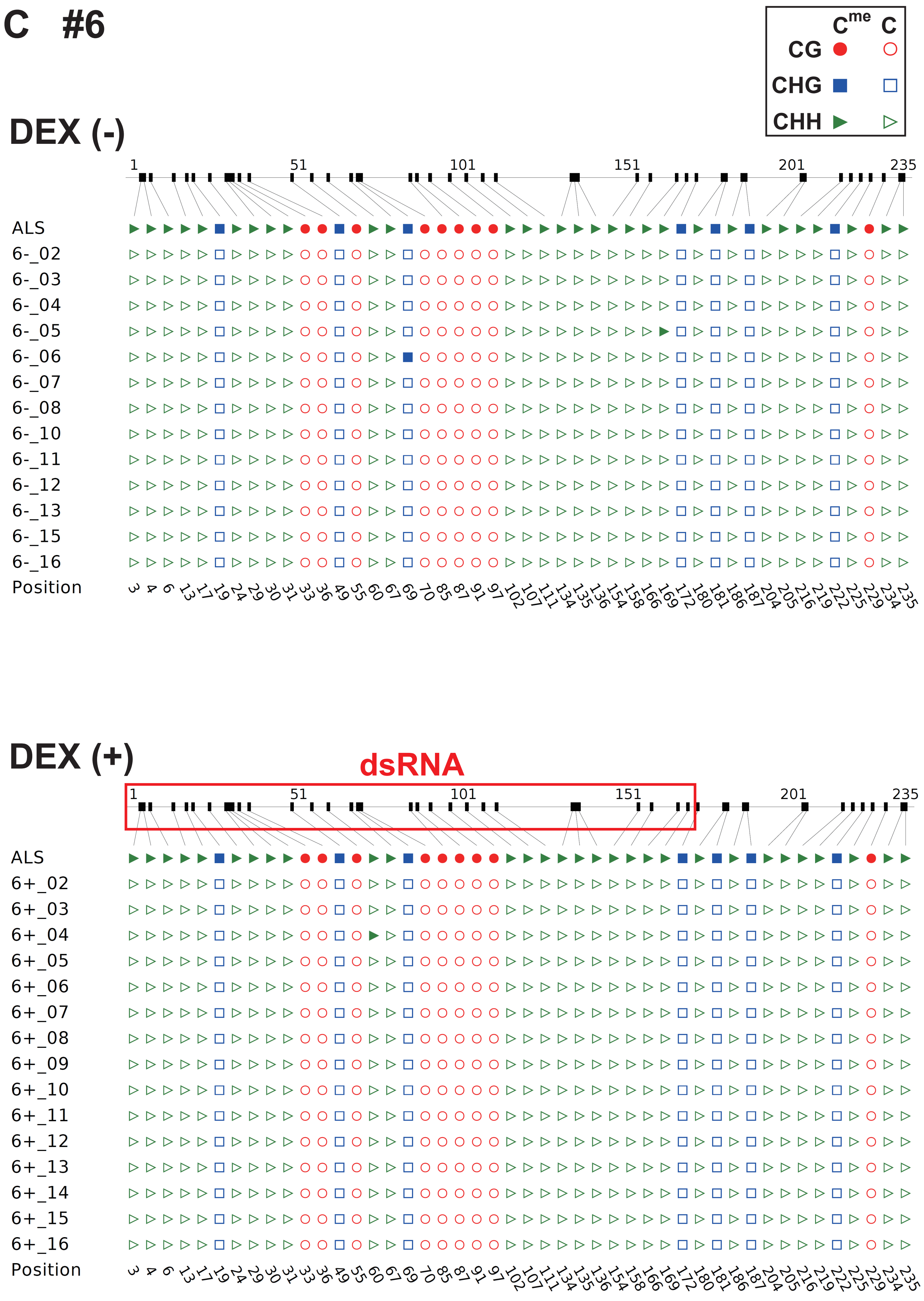
C #6
C^me C
CG
CHG
CHH
DEX (-)
1 51 101 151 201 235
ALS
6-_02
6-_03
6-_04
6-_05
6-_06
6-_07
6-_08
6-_10
6-_11
6-_12
6-_13
6-_15
6-_16
Position
3 4 6 13 17 19 24 29 30 31 33 36 49 55 60 67 69 70 85 87 91 97 102 107 111 134 135 136 154 158 166 169 172 180 181 186 187 204 205 216 219 222 225 229 234 235
DEX (+)
dsRNA
1 51 101 151 201 235
ALS
6+_02
6+_03
6+_04
6+_05
6+_06
6+_07
6+_08
6+_09
6+_10
6+_11
6+_12
6+_13
6+_14
6+_15
6+_16
Position
3 4 6 13 17 19 24 29 30 31 33 36 49 55 60 67 69 70 85 87 91 97 102 107 111 134 135 136 154 158 166 169 172 180 181 186 187 204 205 216 219 222 225 229 234 235

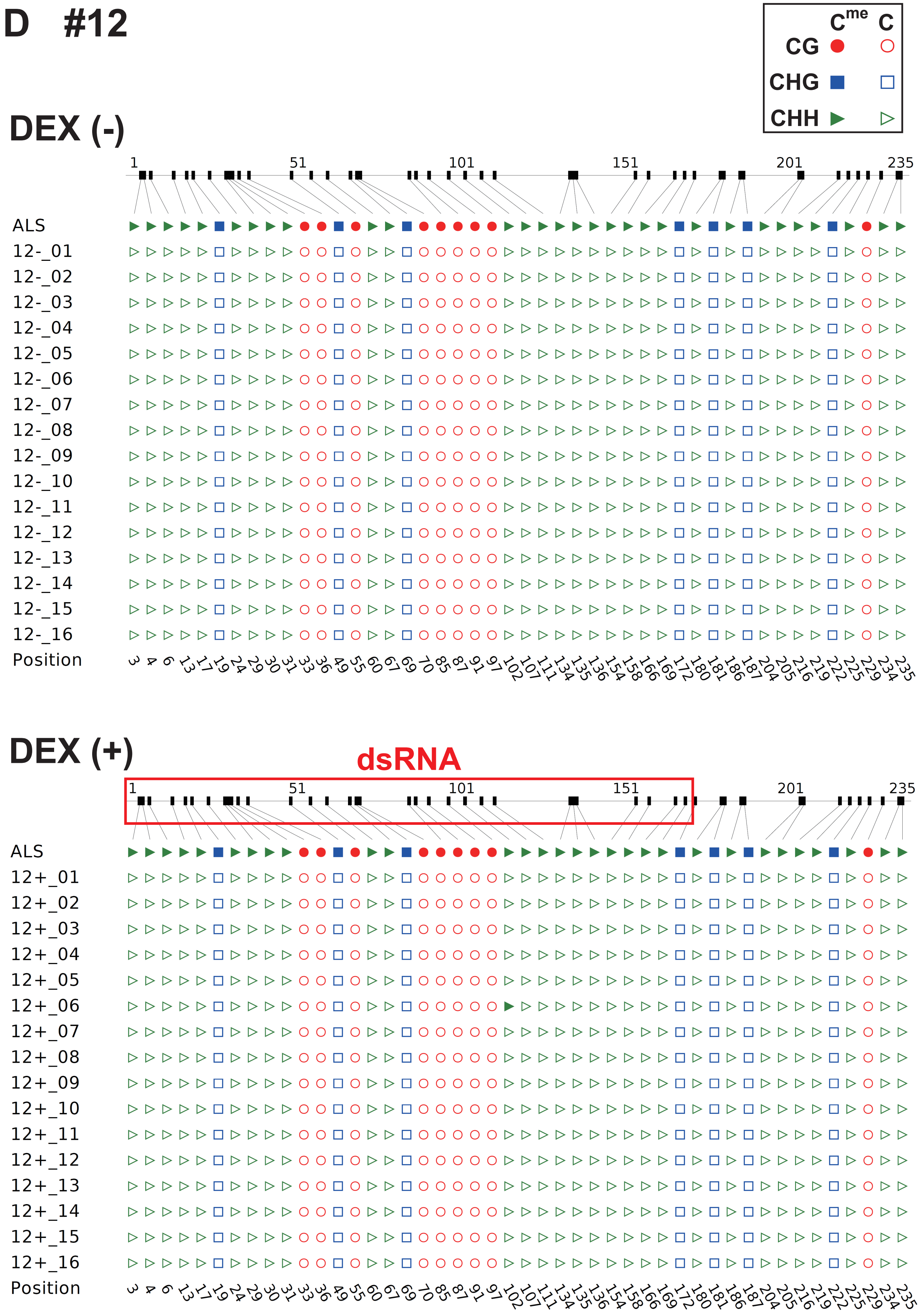

D #12
Cme C
CG
CHG
CHH
DEX (-)
1 51 101 151 201 235
ALS
12-_01
12-_02
12-_03
12-_04
12-_05
12-_06
12-_07
12-_08
12-_09
12-_10
12-_11
12-_12
12-_13
12-_14
12-_15
12-_16
Position
3 4 6 13 17 19 24 29 30 31 33 36 49 55 60 67 69 70 85 87 91 97 102 107 111 134 135 136 154 158 166 169 172 180 181 186 187 204 205 216 219 222 225 229 234 235
DEX (+)
dsRNA
1 51 101 151 201 235
ALS
12+_01
12+_02
12+_03
12+_04
12+_05
12+_06
12+_07
12+_08
12+_09
12+_10
12+_11
12+_12
12+_13
12+_14
12+_15
12+_16
Position
3 4 6 13 17 19 24 29 30 31 33 36 49 55 60 67 69 70 85 87 91 97 102 107 111 134 135 136 154 158 166 169 172 180 181 186 187 204 205 216 219 222 225 229 234 235

Figure S3. Effect of *mALS* siRNA expression on *de novo* methylation of the genomic *ALS* locus in wild-type and transgenic calli. Methylation statuses of the wild-type (WT; A) and the transgenic callus lines #3 (B), #6 (C), and #12 (D) treated with (+) or without (-) DEX for 14 d were analyzed by bisulfite genomic sequencing. Methylated sites are filled symbols for CG sites (red circles), CHG sites (blue squares), and CHH sites (green triangles). The first column (indicated as ALS) is the reference *ALS* sequence and the subsequent columns are the methylation profiles derived from different cells. Red square boxes indicate regions corresponding to the *mALS* dsRNA sequence.
